# Exploring the impact of inoculum dose on host immunity and morbidity to inform model-based vaccine design

**DOI:** 10.1371/journal.pcbi.1006505

**Published:** 2018-10-01

**Authors:** Andreas Handel, Yan Li, Brian McKay, Kasia A. Pawelek, Veronika Zarnitsyna, Rustom Antia

**Affiliations:** 1 Department of Epidemiology and Biostatistics and Health Informatics Institute and Center for the Ecology of Infectious Diseases, University of Georgia, Athens, Georgia, United States of America; 2 Institute of Bioinformatics, University of Georgia, Athens, Georgia, United States of America; 3 Department of Epidemiology and Biostatistics, University of Georgia, Athens, Georgia, United States of America; 4 Department of Mathematics and Computational Science, University of South Carolina Beaufort, Bluffton, South Carolina, United States of America; 5 Department of Microbiology and Immunology, Emory University School of Medicine, Atlanta, Georgia, United States of America; 6 Department of Biology, Emory University, Atlanta, Georgia, United States of America; UNSW Australia, AUSTRALIA

## Abstract

Vaccination is an effective method to protect against infectious diseases. An important consideration in any vaccine formulation is the inoculum dose, i.e., amount of antigen or live attenuated pathogen that is used. Higher levels generally lead to better stimulation of the immune response but might cause more severe side effects and allow for less population coverage in the presence of vaccine shortages. Determining the optimal amount of inoculum dose is an important component of rational vaccine design. A combination of mathematical models with experimental data can help determine the impact of the inoculum dose. We illustrate the concept of using data and models to inform inoculum dose determination for vaccines, wby fitting a mathematical model to data from influenza A virus (IAV) infection of mice and human parainfluenza virus (HPIV) infection of cotton rats at different inoculum doses. We use the model to map inoculum dose to the level of immune protection and morbidity and to explore how such a framework might be used to determine an optimal inoculum dose. We show how a framework that combines mathematical models with experimental data can be used to study the impact of inoculum dose on important outcomes such as immune protection and morbidity. Our findings illustrate that the impact of inoculum dose on immune protection and morbidity can depend on the specific pathogen and that both protection and morbidity do not necessarily increase monotonically with increasing inoculum dose. Once vaccine design goals are specified with required levels of protection and acceptable levels of morbidity, our proposed framework can help in the rational design of vaccines and determination of the optimal amount of inoculum.

## Author summary

An important component of vaccines is the amount of pathogen inoculum, dead or alive, that is included in the vaccine. This inoculum dose, sometimes also referred to as antigen dose, needs to be large enough to induce good protective immunity. However, one usually also wants to keep the dose low to reduce costs, maximize the number of vaccine doses available, and minimize potential vaccine side effects. The inoculum dose is currently chosen based on limited data from clinical trials. In this study, we set up a framework that combines data with mathematical models to illustrate how such a combination could lead to better and more efficient determination of an optimal inoculum dose for vaccines.

## Introduction

Vaccines are the best and most cost-effective defenses we have against many infectious diseases. While the composition of a vaccine can be complex, the most important component is the antigen of the pathogen against which one wants to immunize [1]. Different types of vaccines exist, those based on antigens that contain the pathogen in a non-replicating form, and those that contain the pathogen in a replicating form, usually attenuated to reduce morbidity and mortality [1].

When deciding on the inoculum dose for a vaccine, one often needs to strike a balance between conflicting goals. Higher doses generally lead to more immunity and better protection [2]. Lower doses might reduce vaccine side effects and might also be required if there is a vaccine shortage, for instance, due to a pandemic emergency, manufacturing issues or high costs [3,4]. The ability to predict how changes in inoculum dose impact immune protection and morbidity, and how to achieve the best balance between low dose to reduce costs and side effects and high dose to trigger a robust immune response would significantly contribute toward better vaccine design [5–12].

Currently, the main way to determine vaccine inoculum dose is by trial and error, which is expensive and logistically challenging [13–16]. A way to improve this approach is to combine mathematical models with experimental data. Such approaches are commonly applied to drugs, where pharmacokinetic/pharmacodynamic (PK/PD) models are used in combination with experimental data to optimize drug dosing regimens [17]. Application of a similar approach to vaccines has been recently proposed for tuberculosis [18].

Here, we develop and analyze a quantitative modeling framework that might allow us to eventually predict the optimal inoculum dose for a given vaccine and setting. We develop our modeling framework for live attenuated vaccines using data from two infection experiments, namely influenza A virus (IAV) and human parainfluenza virus (HPIV). We further investigate a scenario for an inactivated vaccine.

Influenza A virus remains a serious health concern. While a vaccine exists, it needs to be reformulated regularly. Even when the vaccine is well-matched to the circulating strain, its efficacy is not as good as that of other vaccines, especially in the elderly. It has been suggested that using a higher inoculum dose in vaccines for this population might be beneficial [19]. Development of a better vaccine that remains protective in the presence of antigenic drift and that has a higher efficacy remains a priority.

Human parainfluenza virus (HPIV) is an important cause of lower respiratory tract illness in children [20–24]. There is currently no licensed vaccine available against HPIV [11,21,24], despite various attempts to develop such a vaccine [25].

While the two pathogens we analyze here are important, the fact that the data we use comes from animal studies means are results are not directly applicable to possible human vaccines. We consider the important contribution of this study to be the development of a conceptual, quantitative framework that may be used to rationally design vaccines and determine an optimal inoculum dose for any pathogen.

## Materials and methods

### Experimental data

We analyzed data from two previously published studies, one on influenza A virus (IAV) infections in mice [26] and the other on human parainfluenza virus (HPIV) type 3 infection in cotton rats [27].

For the IAV study, groups of mice were infected with 6 different inoculum doses of the H1N1 PR8 strain of influenza. Geometric mean viral titers were recorded at different times following the infection with each dose. In addition, lung damage was measured and scored.

For the HPIV study, groups of cotton rats were infected with 5 different doses of HPIV-3. Geometric mean viral titers were recorded at different times following infection in both lung and nose. For the highest inoculum dose, the study additionally reported several virus measurements over the first 96 hours. The study also reported antibody titers 21 days after infection for the 3 lowest inoculum doses for which virus data was reported.

We used an additional data set to estimate a mapping between innate immune response strength and morbidity. This data was taken from a previously reported challenge study of influenza infection in human volunteers [28]. We used the reported values for different components of the innate response (IFN-a, IL6, IL8 and TNF-a) and total symptom score as measure of morbidity.

For further experimental details, we refer the reader to the original studies.

### Mechanistic dynamical infection model

We formulated and implemented a mechanistic, dynamical model of the infection dynamics based on a set of ordinary differential equations. The model is based on our previous work, where we analyzed the relationship between inoculum dose and viral load dynamics [29]. The model is also similar to many other models that have recently been used to model acute viral infections (see e.g. [30–33]).

Our model tracks target cells, virus, and certain immune response components. Uninfected cells, *U*, become infected by free virus, *V*, at rate *b*. Infected cells, *I*, produce virus at rate *p* and die at rate *d*_*I*_. For purposes of comparison with the data, we keep track of dead cells through an extra compartment, *D*.

Free virus infects cells at rate *b*′, is cleared by antibodies at rate $k_{A}^{'}$ or removed due to other mechanisms (e.g. mechanical transport) at rate *d*_*V*_. Note that *b*′ and $k_{A}^{'}$ differ from parameters *b* and *k*_*A*_ to account for experimental units. Since we are modeling short, acute infections, we follow the usual assumption and ignore growth and death of uninfected target cells [30,31].

In addition to the basic infection process, we model components of the innate and adaptive immune response. We consider a generic innate response, *F*, which is produced and decays at rates *p*_*F*_ and *d*_*F*_ in the absence of an infection. Presence of virus leads to an increase in the innate response, with growth leveling off at a maximum rate *g*_*F*_. The virus level at which growth levels off is determined by the parameter *h*_*V*_. The maximum level the innate response can reach is given by the saturation parameter *F*_max_. Since the innate response units are arbitrary, the model is set up such that in the absence of infection, the innate response is at a steady level of *F* = 1, which leads to *p*_*F*_ = *d*_*F*_. We fix the parameter representing the decay rate at *d*_*F*_ = 1 per day, which is in line with estimates from an influenza infection analysis in ponies [34].

The innate response is modeled as having two main mechanisms of action. First, it can directly counteract the virus by reducing virus production rate of infected cells [35]. In our model, the strength of production suppression is determined by the parameter *s*_*F*_. The second action of the innate response is to induce the adaptive response, as described next.

For the adaptive response, we focus on B-cells and antibodies, which are the major correlates of protective immunity for most vaccines, including HPIV and IAV [23,36]. The dynamics of activated B cells is modeled as increasing in a sigmoidal manner dependent on both the amount of virus (antigen) and the innate response, with a maximum rate *g*_*B*_. The parameter *h*_*F*_ determines the level of virus and innate response at which B-cell growth saturates. Since we are focusing on the short-term dynamics of the system, B-cell decay is ignored. In the absence of an infection, B-cells are set to an arbitrary level of 1. B-cells produce antibodies at rate *r*_*A*_. Antibodies decay naturally at rate *d*_*A*_ and bind to and remove free virus at rate *k*_*A*_.

The model is implemented as a set of ordinary differential equations given by the following set of equations:

$$\begin{array}{lll} \text{Uninfected}\;\text{cells} & \dot{U} & =-bUV\\ \text{Infected}\;\text{cells} & \dot{I} & =bUV-d_{I}I\\ \text{Dead}\;\text{cells} & \dot{D} & =d_{I}I\\ \text{Virus} & \dot{V} & =\frac{p}{1+s_{F}F}I-d_{V}V-k_{A}^{\prime }AV-b^{\prime }UV\\ \text{Innate}\;\text{response} & \dot{F} & =p_{F}-d_{F}F+\frac{V}{V+h_{V}}g_{F}\left(F_{max}-F\right)\\ \text{B}\;\text{cells} & \dot{B} & =\frac{FV}{FV+h_{F}}g_{B}B\\ \text{Antibodies} & \dot{A} & =r_{A}B-d_{A}A-k_{A}AV \end{array}$$ (1)

### Model fitting

The model is fit to the IAV and HPIV data. For IAV the fit is to the virus load and lung damage data. For HPIV, the fit is to the virus load and antibody data. For each pathogen, we fit the model to data for all different inoculum doses simultaneously. For each inoculum dose, *i*, we estimate the starting value for the virus inoculum, *V*_*i*_. All other model parameters are shared across different inoculum doses.

Model performance is assessed by the sum of squared residuals (SSR). To allow computation of a single SSR value for the different experimental variables, the contribution of each variable is non-dimensionalized by dividing by the variance of the data. To give the different experimental variables comparable importance, we divide each variable by the number of data points. This amounts to over-weighting the few data points for lung damage (IAV) and antibody response (HPIV) and reducing the weight for the more plentiful viral load data. Mathematically, the expression for the SSR is given by

$$SSR=\sum_{i,t}\frac{1}{N_{V^{d}}}\frac{(V_{i,t}^{m}-V_{i,t}^{d}{)}^{2}}{\sigma^{2}(V^{d})}+\frac{1}{N_{X^{d}}}\frac{(X_{i,t}^{m}-X_{i,t}^{d}{)}^{2}}{\sigma^{2}(X^{d})}$$

Here, *V* is viral load (on a log scale), and *X* represents either antibodies (for HPIV) or damage (for IAV), the superscript indicates model (*m*) or data (*d*), the sum runs over all inoculum doses, *i*, and all time points, *t*. *N* indicates the number of data points for either the virus or the other variable, *σ*^2^ indicates the variance for that variable. Since both damage and antibodies (*X*) are measured in different units in the data and the model, each are normalized before subtracting and squaring.

The experimental data for virus load is quantified in hemagglutination units and expressed in virus per lung (IAV), and plaque forming units per gram of lung (HPIV). We assume that the virus variable in our model is in the same units. For damage, the experimental data is reported as percent of lung area that is damaged/destroyed. For IAV, both the model-predicted damage (number of dead cells) and the damage as reported in the data is divided by the maximum value of model prediction or data measurements (for the whole dataset, across all inoculum doses). This leads to a re-scaling of both quantities, thus allowing one to compare model and data. We thus assume that damage as tracked by the model is proportional to damage reported in the data. The same approach is used for the antibody data for HPIV, where the model predictions are ‘antibodies in the system’ while the experimental data are antibodies based on a plaque reduction neutralization assay. The re-scaling again allows for fitting, with the assumption that the model and experimental quantities are proportional.

While this re-scaling is only necessary for the instances where we compare model and data (lung damage for IAV and antibodies for HPIV), for consistency between models, we plot re-scaled values for both lung damage and antibodies for both IAV and HPIV.

The model is being fit by varying model parameters to minimize the *SSR*. When doing so, we take into account left-censored nature of the data. If the reported virus load is at or below the limit of detection (LOD, which is 0.27 log10 units for IAV and 2 log10 units for HPIV as reported in the original studies), and the model prediction is below the data, we do not count any difference between model and data for any model prediction [37,38].

### Model implementation

All computations were done in the R programming language version 3.4.3 [39]. Fitting was done using the nloptr optimizer package [40], differential equations were integrated using the deSolve package [41]. All data and code required to reproduce all results presented here are supplied in S1 Code.

## Results

### Data extraction

The data used for our study was obtained from the original reports as follows.

For the IAV study, we obtained log viral load and lung lesion score expressed in percent lung damage from table 1 of [26]. The viral kinetics of the highest inoculum dose strongly hints at survivor bias (see figure 1 of [26]). Specifically, the data suggest that sicker mice, with presumably higher virus load, were killed and sampled first, while less sick mice, with presumably lower virus load, were kept alive and sampled later. We, therefore, decided to exclude the data for the highest inoculum dose from consideration, leaving us with viral load and percent lung damage data for 5 different inoculum doses.

**Fig 1:**
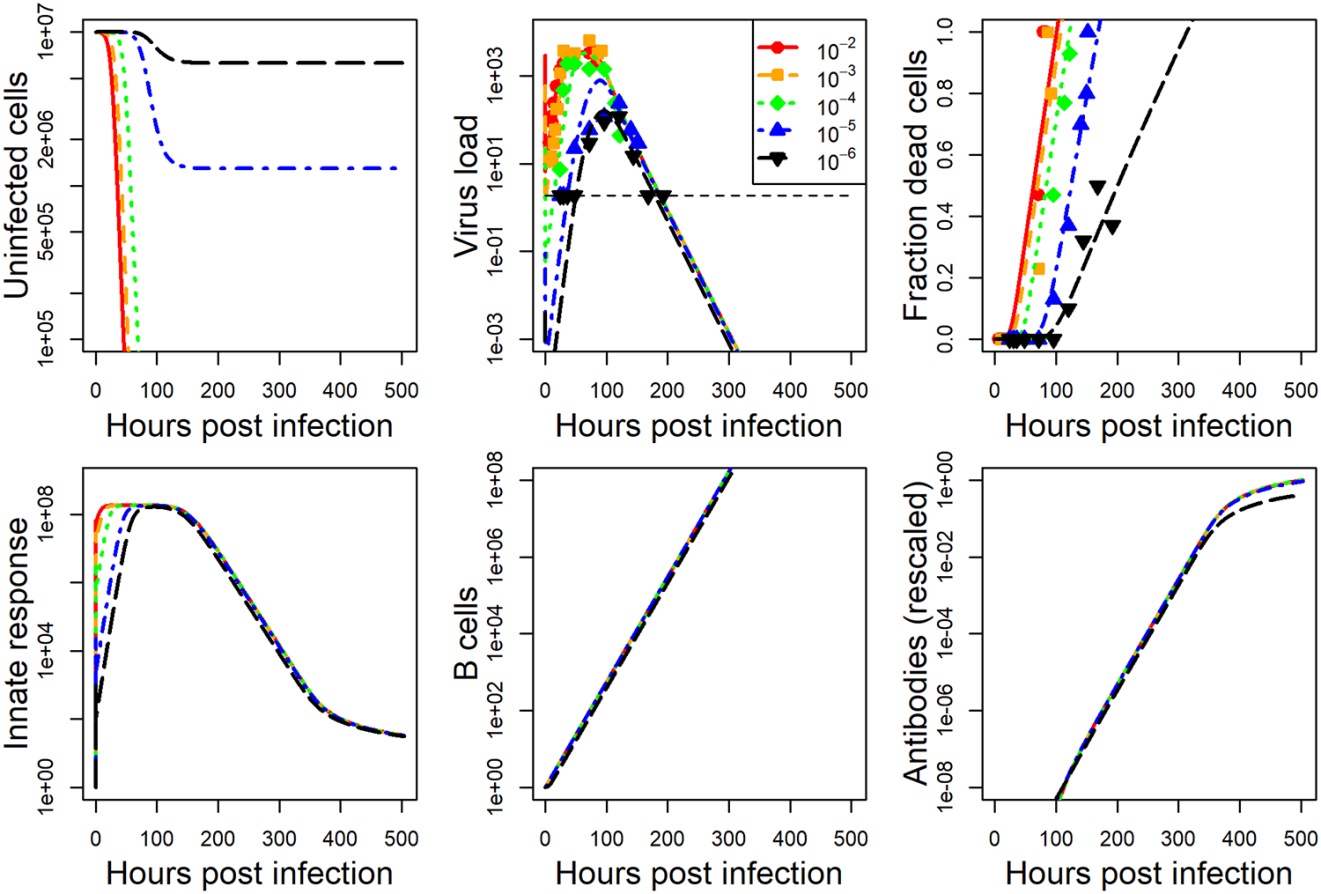
Fit of model to IAV infection data at five different inoculum doses. Kinetics for 6 of the seven model compartments for the best fit model are plotted. Since infected cells kinetics very closely follows virus kinetics, we did not plot it. Dashed horizontal line indicates the limit of detection for virus load. Best fit parameter values are provided in S1 Text. Data was available for virus load and cell damage. Virus load data was reported as hemagglutination units and cell damage was reported as percent lung cells that are pathological. Model virus load is in the same units as the experimental data. All other model quantities are in units of numbers. Damage in the model is measured as number of dead cells. Both dead cells and antibodies are rescaled as described in the method section to allow comparison between model and data. The plot shows the rescaled quantities.

For the HPIV study, we focused on lung viral load. The data was extracted from figures 1 and 2 of [27] using Engauge Digitizer [42]. Viral load kinetics for the highest inoculum were measured twice with some overlap in times (24h and 96h). We averaged data for these times from the 2 experiments. We additionally obtained data on antibody titers for those inoculum doses for which viral load was reported (the 3 lowest inoculum doses) from figure 3 of [27].

For the data linking innate response to symptoms, total symptoms score data was extracted from figure 3 and innate immune response data from figures 5 and 6 of [28] using Engauge Digitizer. More details on how this data was used are provided in other parts of this paper. The data are part of S1 Code.

### Model development and fitting

**Fig 2:**
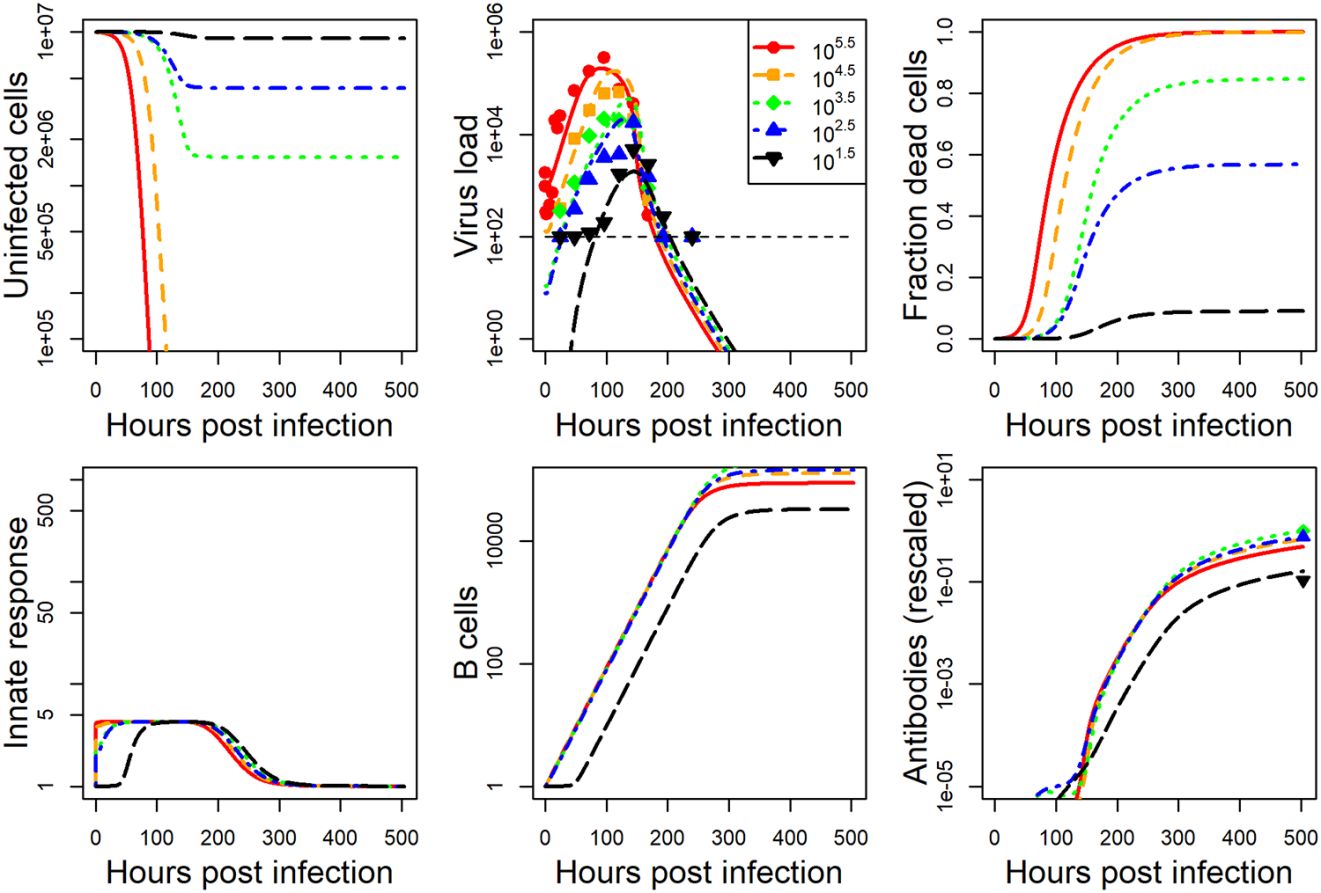
Fit of model to HPIV infection data at five different inoculum doses. Kinetics for 6 of the seven model compartments for the best fit model are plotted. Since infected cells kinetics very closely follows virus kinetics, we did not plot it. Dashed horizontal line indicates the limit of detection for virus load. Best fit parameter values are provided in S1 Text. Data was available for virus load and some antibody levels. Virus load data was reported as plaque forming units per gram of lung and antibody titer was reported as plaque reduction neutralization units. Model virus load is in the same units as the experimental data. All other model quantities are in units of numbers. Damage in the model is measured as number of dead cells. Both dead cells and antibodies are rescaled as described in the method section to allow comparison between model and data. The plot shows the rescaled quantities.

The model we developed is described in detail in the methods section. For each data set, we fit the model to the viral load data, and either lung damage (IAV) or antibody (HPIV) data. Details on the fitting approach are provided in the methods section. The model fits and data for IAV and HPIV are shown in Figs 1 and 2 respectively. Parameter values for the best fits are given in the S1 Text.

While the dynamics for the 2 pathogen-host systems look broadly similar, it is noticeable that the IAV infection leads to a stronger innate response and more host damage (fraction dead cells). This is a particular feature of the influenza strain used in those experiments. Different influenza strains in mice, and certainly most influenza infections in humans, do not cause a large amount of cell damage—though some of the more serious influenza infections, e.g. with the H5N1 strain, can lead to a large amount of damage.

### Quantifying immune protection

We want to quantify the amount of protective immunity induced by different inoculum doses. We focus on the B-cell and antibody component of the adaptive immune response. Provided antibodies are specific to the pathogen, higher levels of antibodies generally lead to better protection [43–45]. Recent studies for influenza vaccines [46,47] demonstrated that the following function provides a good mapping from antibody titer to the level of protection from infection:

$$P=1-\frac{1}{1+e^{k_{1}(log(A)-k_{2})}}$$ (2)

**Fig 3:**
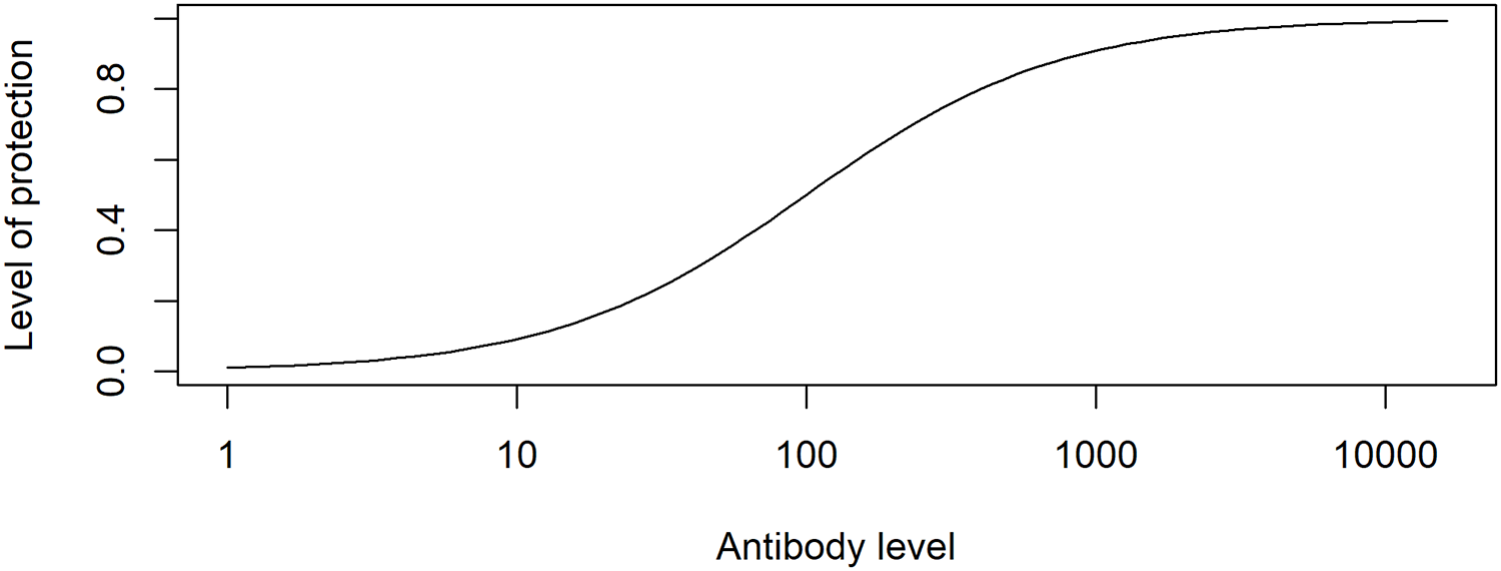
Protection as function of antibody levels (k_1_ = 1, k_2_ = log(100)). Protection is the fraction of individuals in a population who are protected by the vaccine for a given level of antibodies. Antibody levels are on an arbitrary unit.

Here, the level of protection, *P*, varies between 0 and 1, with low protection for low levels of antibody titer, *A*, and maximum protection at high levels. The constants *k*_1_ and *k*_2_ determine the slope of the curve and the level at which protection is at 50% respectively (see [46] for more details). This functional shape is also consistent with data for other pathogens [43–45]. Fig 3 illustrates this relationship between antibody levels and protection graphically.

Our model represents antibodies in units of numbers of antibodies. In general, experimental studies report antibody neutralizing titers or similar assay-specific units. For this reason, and because we have no data for the correlation between antibody titers and protection for either the HPIV or IAV data we analyze, it is impossible to determine specific choices for *k*_1_ and *k*_2_ for our study systems. We instead chose values such that the antibody levels considered span the full range from low to high protection levels. Specifically, we set *k*_1_ = 1 and *k*_2_ = *E*(*log*(*A*)) where *A* is the range of antibody levels predicted by our model for different inoculum doses and *E* is the expected value. This choice is essentially arbitrary and therefore the protection curves we present below are to be understood conceptually.

### Quantifying morbidity

It is still not fully understood how virus and immune response affect host morbidity, i.e. the severity of symptoms. For virus infections, host morbidity can be due to virus-induced death of infected cells, as well as immune response mediated pathology. A study of influenza infection in humans suggested that a model in which symptom score was proportional to innate cytokine levels provided an adequate fit to the data [48]. Another study of influenza infections used a combination of innate cytokine (interferon) levels and cell death to define morbidity as *M* = *D* + (1 − *D*)*g*(*F*), where *D* is the total number of dead cells, and *g*(*F*) was chosen to be a sigmoidal mapping of log interferon levels [49]. Similarly, a previous model for dengue infections assumed that morbidity was proportional to the peak of the innate response, i.e. *M* ~ *max*(*F*) [50]. In the case of vaccines, strong pathological effects such as the death of a meaningful fraction of target cells do not occur. It therefore seems most reasonable to express morbidity (strength of symptoms) as a function of the innate immune response.

**Fig 4:**
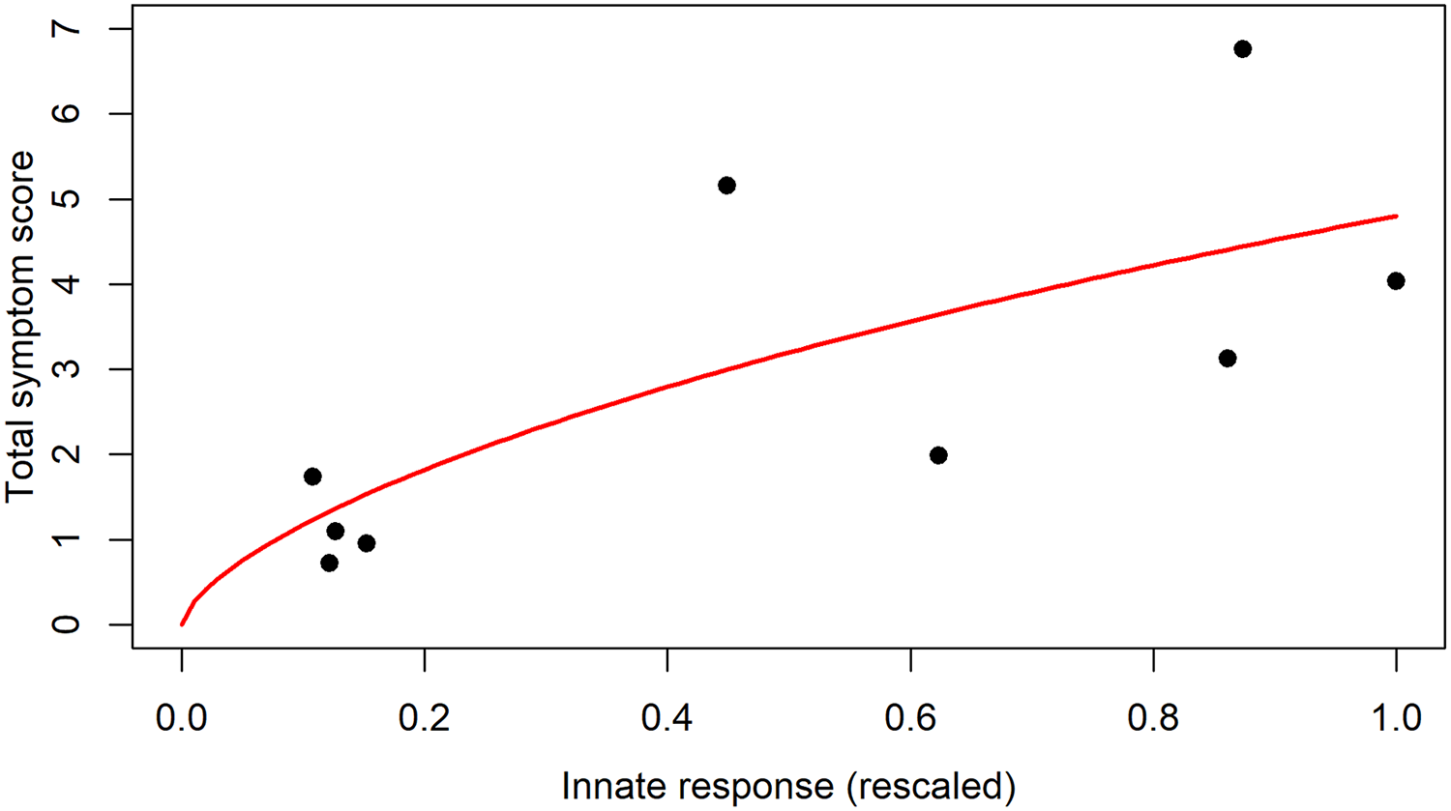
Data and best fit model for the connection between immune response and symptoms. The innate response is the scaled sum of IFN-a, IL6, IL8, and TNF-a, symptoms are the total symptom score, both quantities are from [28]. The line shows the best fit provided by the equation mapping innate response to symptoms/morbidity.

To obtain an estimate for a mapping between innate immune response and morbidity, we use data from a previously reported challenge experiment of influenza infection in human volunteers [28]. We use the reported values for different components of the local innate response (IFN-a, IL6, IL8, and TNF-a) and, after scaling each component to a maximum value of 1, sum them to obtain an estimate for the total innate response strength. This total response quantity is again scaled and then mapped to morbidity, measured as total symptom score. Fig 4 shows the data and the best fit of a sigmoidal model that provides a mapping between innate response and morbidity.

The model is given by

$$M=\frac{aF^{c}}{b+F^{c}}$$ (3)

where *M* is morbidity as measured by total symptom score, and *F* is the scaled innate response. Best fit parameter values are *b* = 6.5 and *c* = 0.66. The parameter *a* was fixed at 36, corresponding to the maximum score possible based on the study protocol [28]. While a simpler linear model would fit the data equally well, it is less biologically reasonable since it would allow an unbounded increase in symptoms.

From our simulations, we obtain the time course of the innate response, *F*, for each inoculum. After rescaling this quantity, i.e. dividing by the maximum of *F* across all doses, we use Eq (3) to compute the time course for morbidity, *M*. Finally, we take the integral of the morbidity to compute total morbidity as the area under the morbidity curve (MAUC), given by:

MAUC=∫M (4)

The integral goes over the duration of the infection. Since this approach mixes model simulations based on animal infections with morbidity estimates based on human data, the resulting morbidity curve should be interpreted in a similar conceptual way as the protection curve described above.

### Immunity and pathogenesis as function of inoculum

**Fig 5:**
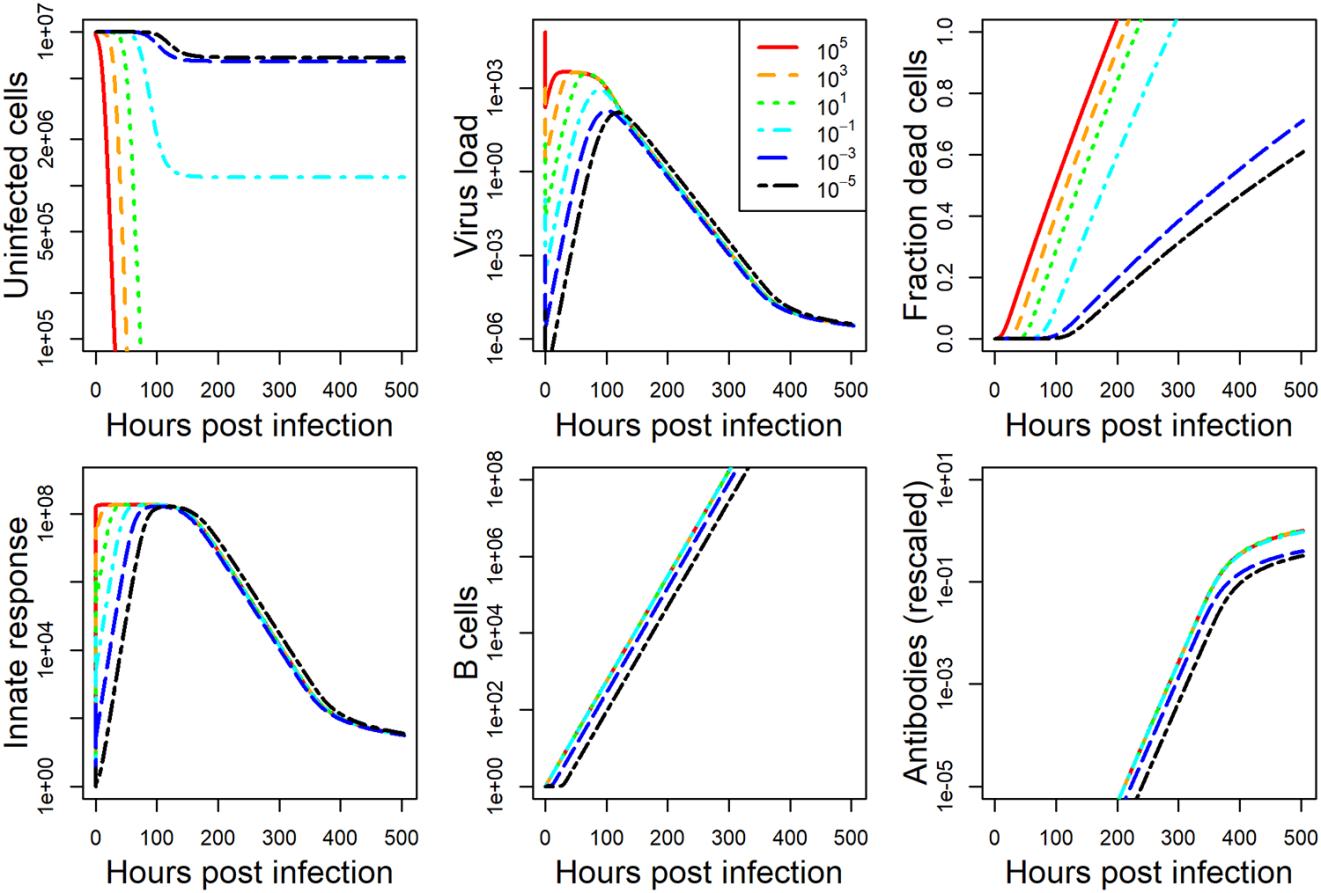
IAV model simulation for a range of inoculum doses. All plot settings are the same as described for Fig 1.

**Fig 6:**
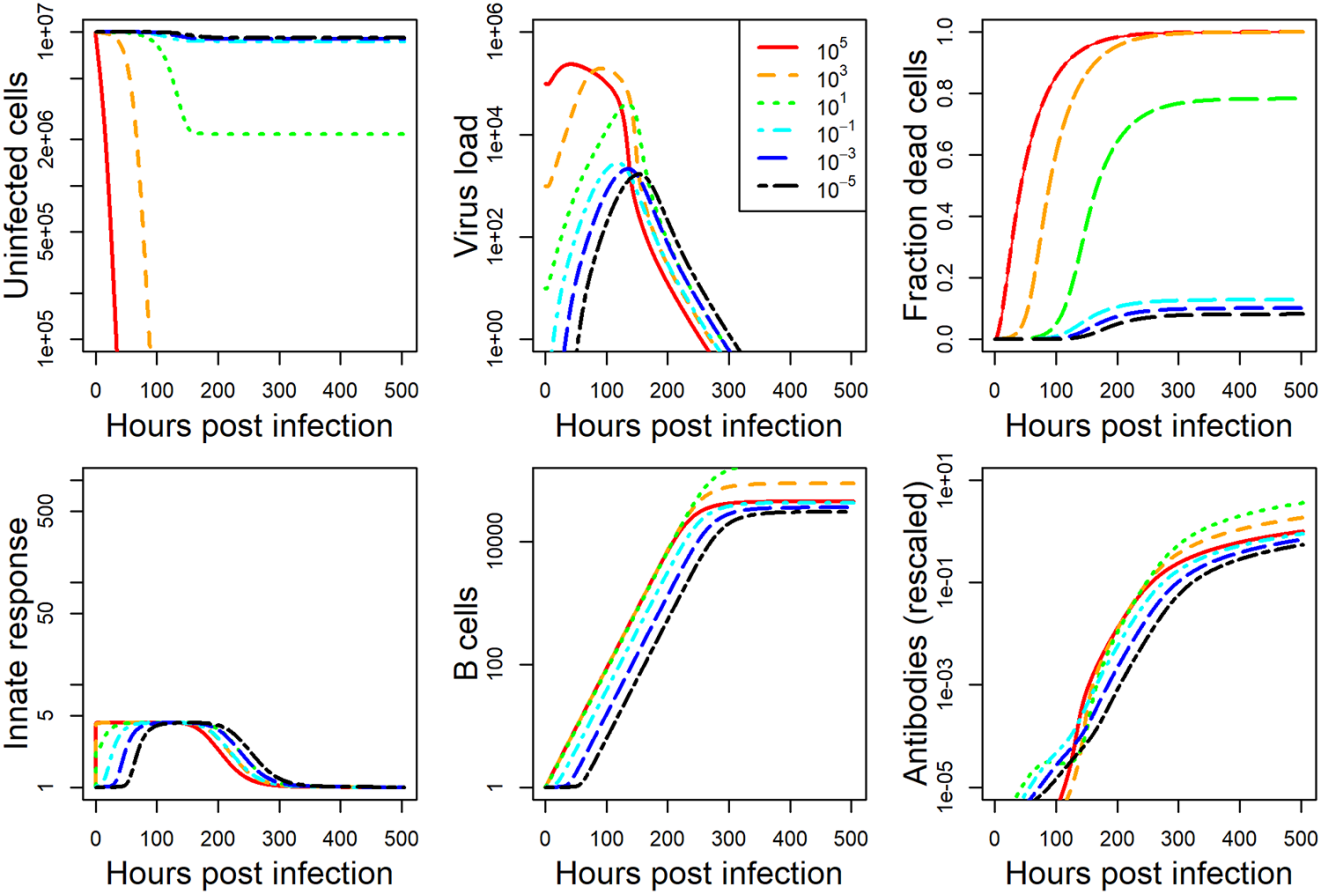
HPIV model simulation for a range of inoculum doses. All plot settings are the same as described for Fig 2.

After fitting the dynamical infection model (1) to each data set, we used the best-fit parameter values and ran simulations for a range of inoculum doses. Several time-series for the IAV and HPIV model simulations spanning the whole range of simulated inoculum doses are plotted in Figs 5 and 6.

The model fit to IAV shows increase in the innate response as inoculum dose increases, and an increase, followed by a leveling off, for the antibodies. For HPIV, we see the innate response increasing more rapidly as dose increases but reaching the same saturating level. Antibodies initially increase, peak at intermediate inoculum dose, and then decrease.

**Fig 7:**
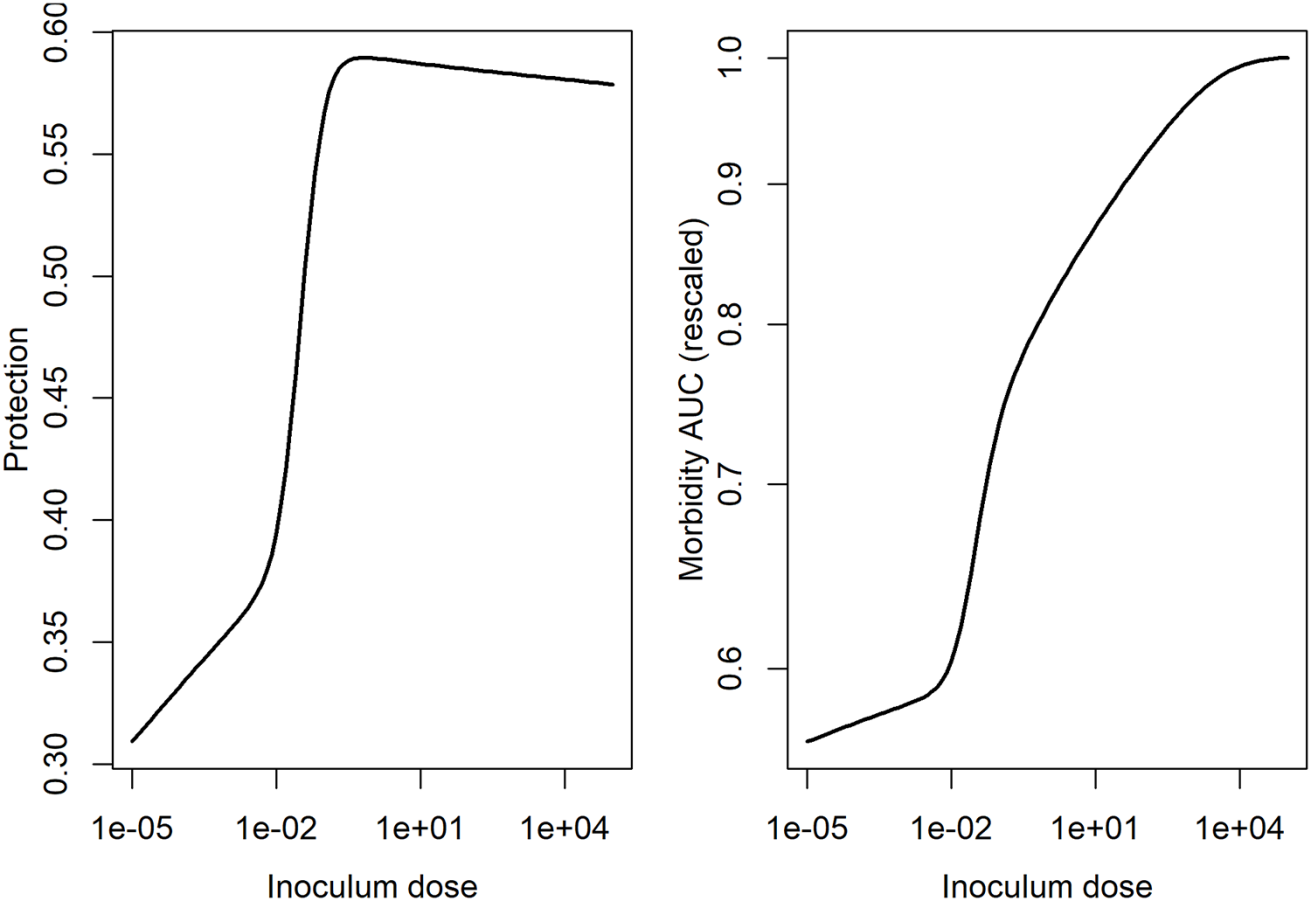
Inoculum dependent protection and damage for the IAV infection model. Protection was determined based on antibody levels predicted by the model and computed using the equation for protection, P(A), described above. Morbidity was determined from the innate response levels predicted by the model and computed using the MAUC equation described above. To allow for better comparison with protection, we also scaled the MAUC values by their maximum.

**Fig 8:**
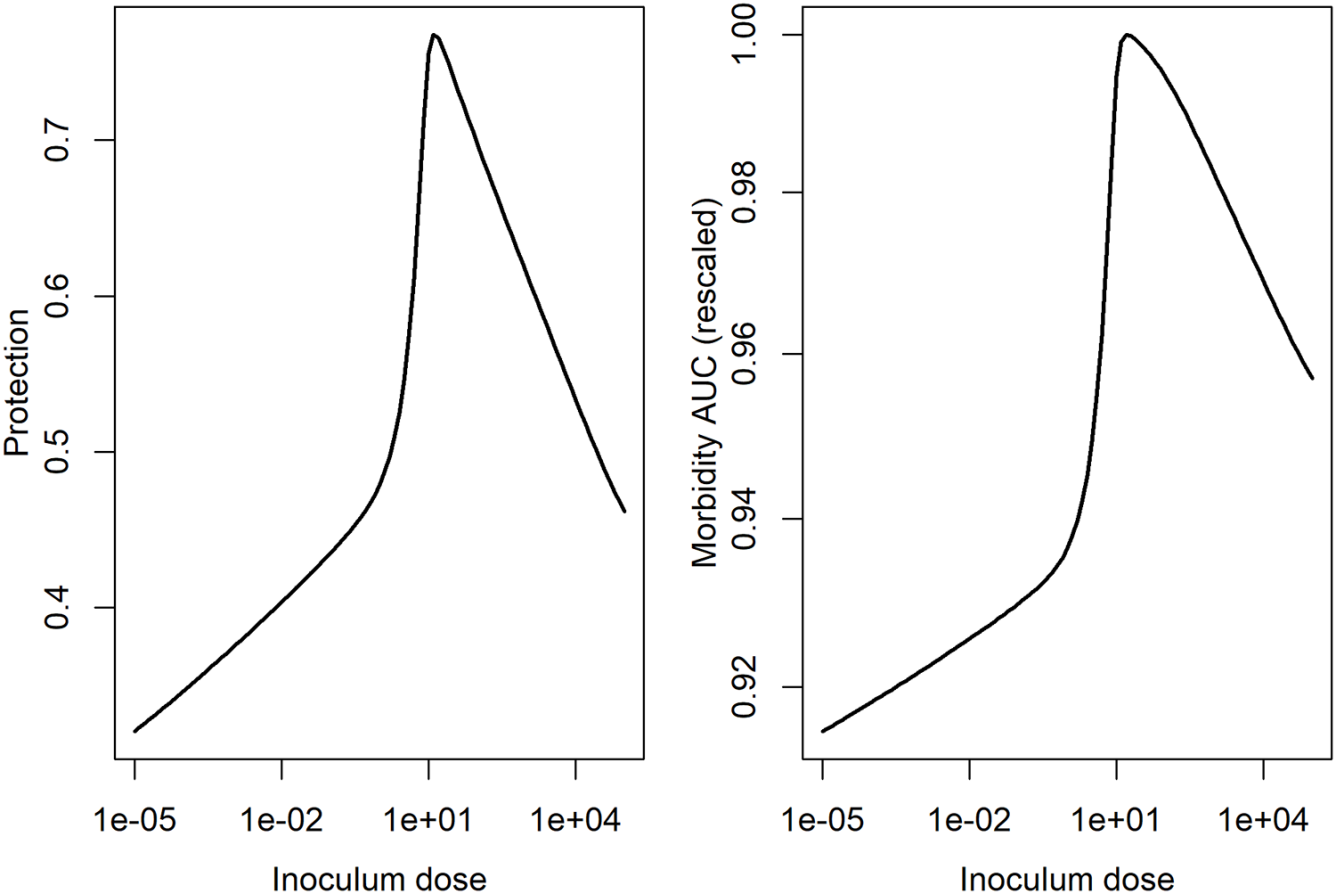
Inoculum dependent protection and damage for the HPIV infection model. Protection was determined based on antibody levels predicted by the model and computed using the equation for protection, P(A), described above. Morbidity was determined from the innate response levels predicted by the model and computed using the MAUC equation described above. To allow for better comparison with protection, we also scaled the MAUC values by their maximum.

From these time-series, the level of protection and morbidity was computed. The model is simulated for 21 days, predicted antibodies are recorded at the final time. From these antibody levels, we compute immune protection using Eq (2). We also record the predicted innate immune response, and, after scaling, use Eq (3) to compute morbidity, and by integrating the area under the curve, determine the total amount of morbidity during the infection. Those results are shown for IAV in Fig 7 and for HPIV in Fig 8.

We find for these examples that protection initially increases with inoculum dose, followed by a slight (IAV) or strong (HPIV) decrease for very high doses. Morbidity increases for IAV through the whole range, and shows the same “increase, then decrease” pattern as HPIV.

### An inactivated vaccine model

The model and data above are for replicating pathogens, as such representing live, attenuated vaccines. Another important category of vaccines are those where the pathogen is killed and non-replicating. A modification of the above model can be used to simulate such a vaccine. For such a vaccine, cells do not get productively infected, and one can remove the variables tracking uninfected and infected cells. The model simplifies to

$$\begin{array}{lll} \text{Virus}\;\text{antigen} & \dot{V} & =-d_{V}V-k_{A}^{\text{'}}AV\\ \text{Innate}\;\text{response} & \dot{F} & =p_{F}-d_{F}F+\frac{V}{V+h_{V}}g_{F}\left(F_{max}-F\right)\\ \text{B}\;\text{cells} & \dot{B} & =\frac{FV}{FV+h_{F}}g_{B}B\\ \text{Antibodies} & \dot{A} & =r_{A}B-d_{A}A-k_{A}AV \end{array}$$

**Fig 9:**
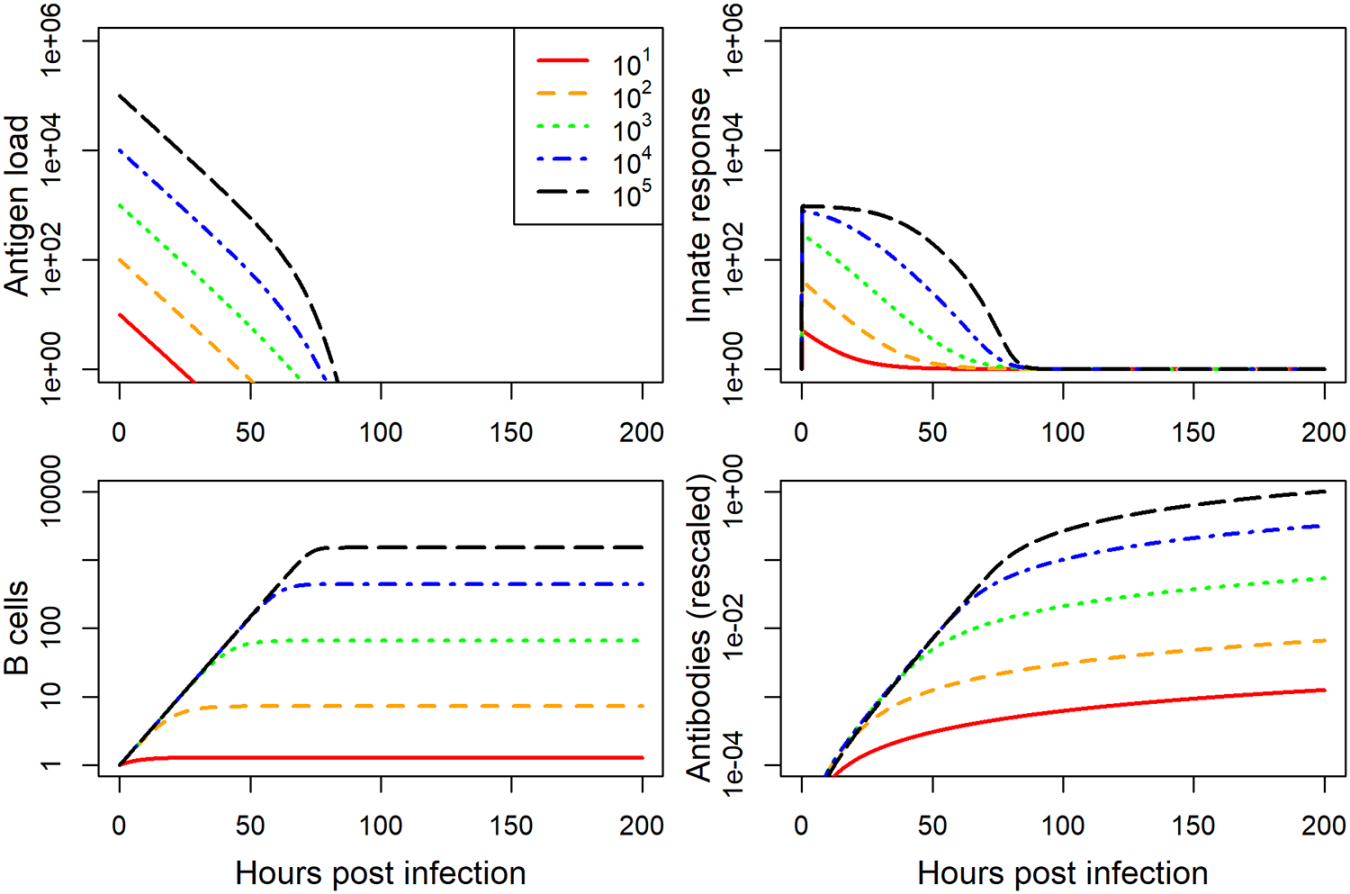
Model for non-replicating vaccine. Model parameters were set to d_V_ = 0.1, $k_{A}^{'}=1E-5$, h_V_ = 1E5, g_F_ = 1E3, F_max_ = 1E3, h_F_ = 100, g_B_ = 0.1, r_A_ = 1, d_A_ = 1E − 6, k_A_ = 1E − 6. Initial conditions are F = 1, B = 1, A = 0 and varying values for antigen load.

**Fig 10:**
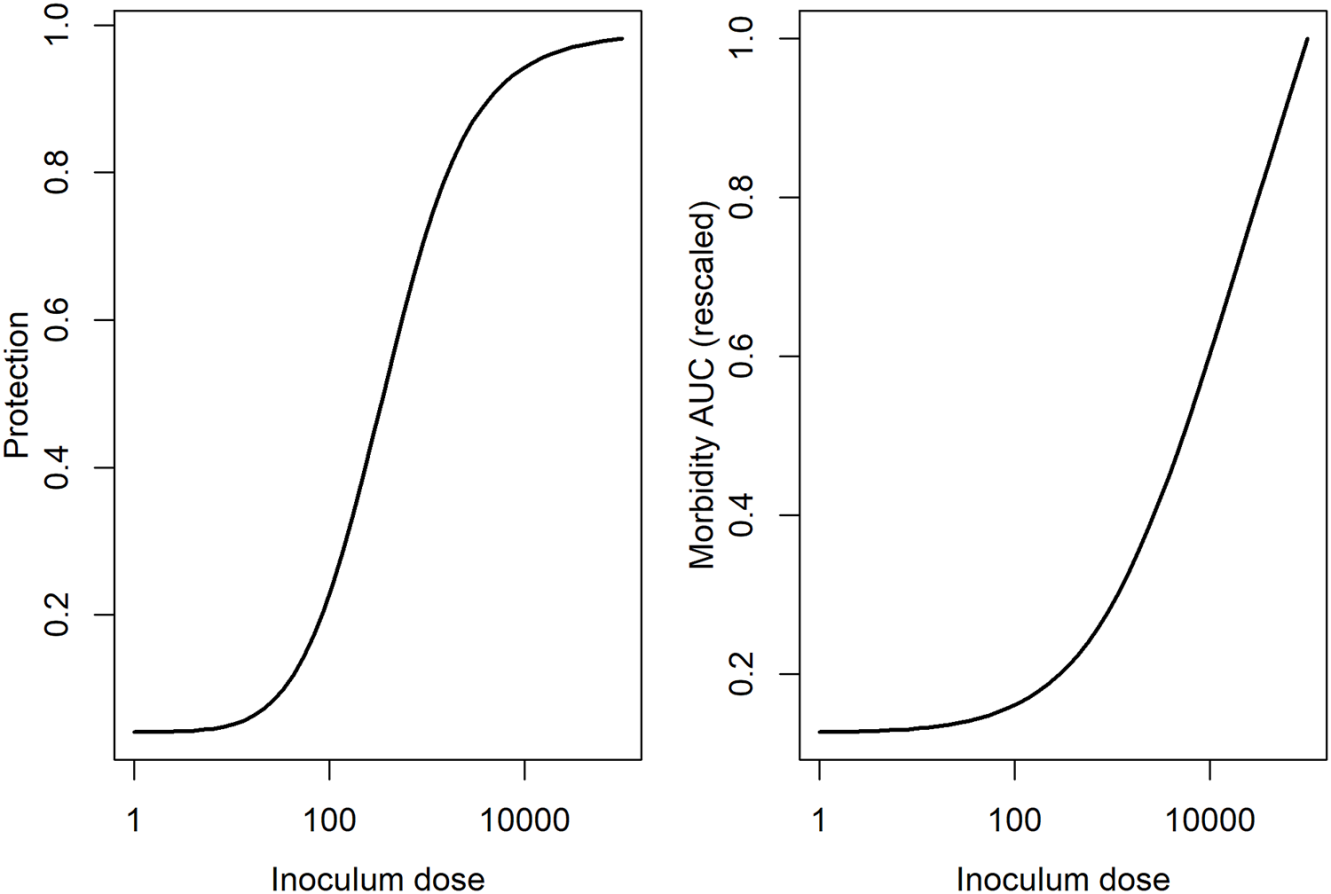
Inoculum dependent protection and damage for the inactivated vaccine model.

We were not able to find data in the published literature that was detailed enough to allow model fitting. We therefore instead chose values for model parameters that produced reasonable dynamics and explored the impact of inoculum/antigen dose on protection and morbidity for such a generic model. Fig 9 shows simulated time-series for different inoculum doses, Fig 10 the resulting predicted immune protection and morbidity.

### Determining an optimal inoculum dose

For any given vaccine, one wants to find out the inoculum dose which is “optimal”. There is no single definition of “optimal”. One important criterion is to get immune memory and thus protection as high as possible. Another criterion could be to achieve high protection with as few side effects (morbidity) as possible. Yet another criterion could be to achieve maximum protection at the lowest possible dose to allow vaccination of as many individuals as possible in the presence of vaccine shortages. These different targets lead to a general optimization problem, where the objective is to find a dose *D* that maximizes a function *f*(*D*) which is made up of targets for each criterion. For instance a simple hypothetical function to be maximized could be *f*(*D*) = *b*_1_ *P*(*D*) + *b*_2_ *M*(*D*), where *b*_1_ is a positive parameter weighing the criterion of protection, and *b*_2_ is a negative parameter weighing the criterion for morbidity. Another function could be a min-max scenario, where one tries to find a range bounded by the minimum dose that still provides some critical level of required protection and the maximum dose above which morbidity is above an acceptable level.

For illustrative purposes, we show results for one such hypothetical objective function *f*(*D*), namely the ratio of protection and morbidity, in S1 Text. This is purely illustrative and not meant to suggest this as a particularly useful choice of criteria. The specific criteria will be different for different pathogens and scenarios. For instance for a very severe disease in an outbreak setting (e.g. Ebola), the goal could be to produce vaccines at the lowest required dose to achieve some critical level of protection, without worrying much about side effects. In contrast, routinely given childhood vaccines where shortage is not a problem will need to focus on the balance between protection and side-effects, likely without too much emphasis on dose sparing to stretch vaccine supply.

## Discussion

In this study, we used a combination of data and models to explore how inoculum dose impacts immune protection and morbidity. We found that for the examples investigated, sometimes there is a monotonic or almost monotonic increase of protection and morbidity as inoculum increases (inactivated vaccine and IAV examples), while other times protection and morbidity can decline once dose increases beyond some value (HPIV example). In this latter scenario, we found with our model that once inoculum doses increase beyond some threshold, the innate immune response is triggered so strongly that it leads to quick pathogen clearance, which in turn leads to a reduced activation of the adaptive immune response and thus reduced immune protection.

If or how this pattern shown in our model applies to real settings is unclear. In many situations, a higher inoculum dose of either live attenuated or killed antigen in a vaccine leads to a stronger immune response and subsequently likely better antibody or T-cell mediated immune protection [51]. We are not aware of a clear example showing increased inoculum dose of an attenuated or killed vaccine leading to reduced immune protection, however, our model and general biological mechanisms suggests it as a possibility.

We want to re-emphasize that our analysis presented here should be understood as the development of a conceptual framework towards the use of models and data for potential vaccine optimization. Several obvious limitations do not allow one to consider this study more than conceptual. The data we had available are reported as averages of multiple animals, the hosts being studied (mice and cotton rats) are not natural hosts for the pathogens they were infected with, we combined some human data with the animal data, and the relative sparsity of the data does not allow us to fully validate our model. We built and used a model that we believe can be justified based on our biological understanding of the processes happing during such acute infections. However, our model is too complex for the data at hand and leads to overfitting. We chose to use this model based on biological grounds. There are likely many alternative, biologically reasonable, model formulations which might lead to different results, again emphasizing the conceptual nature of our work.

We believe that using an approach that combines modeling with data can help in the development of more efficient vaccines. The key toward that goal is the availability and integration of the right kind of models and data. Data should be collected for correlates of immune protection (e.g. specific antibodies, B-cells, T-cells) and direct or indirect markers of morbidity (e.g. symptom score, side-effects) for a few different inoculum doses. Further measurements that can help validate models, e.g. pathogen load over time, might be also be useful. Similarly, data that measures risk of infection at several different levels of the correlates of protection will be needed. Combining those data with models would allow testing and calibration of a model, which could then be used to produce predictions for protection and morbidity over a full range of inoculum doses, including those not experimentally measured.

Being able to predict the expected protection achieved for a given inoculum dose can help in the design of vaccines in cases when only limited antigen is available, e.g. in emergency situations [4]. Having information on both immune protection and expected morbidity allows one to determine an optimal inoculum dose based on the—often conflicting—goals of high protection and low morbidity. For instance, one could systematically answer questions such as, “If we require at least 80% immune protection, what would the minimum amount of inoculum need to be? And what level of morbidity/side-effects would this induce?” Currently, both modeling and experiments are not yet able to be used in such a specific manner. However, a tighter integration of experiments with models, and further model refinement should allow one to use the modeling approach discussed here in the future to help design vaccines.

Some promising extensions and refinements of the models are inclusion of further components of the immune response. For instance, given that T-cells are also known to play an important role in immune protection and are affected by inoculum dose [52,53], it would be beneficial to extend experimental and modeling studies in the future and consider both the B-cell and T-cell components of the adaptive response. Similarly, provided more detailed data on specific components of the innate response is available, including those components explicitly in the models might be useful. Another extension would be to consider stochastic models, which would better be able to capture variation among patients. This would require individual host data to be available for analysis and modeling.

Our study fits into the recently proposed framework of Immunostimulation/Immunodynamic (IS/ID) modelling, which has been proposed as a framework to combine models and data for better vaccine formulation decisions [18], in analogy to the well-established pharmacokinetic/pharmacodynamic (PK/PD) modelling approach widely used in drug development [17].

To summarize, we developed a modeling framework that might allow a systematic and quantitative determination of the impact of different inoculum doses on resulting immune protection and morbidity. We applied this approach to several data sets to illustrate how the general concept can lead to important insights, e.g. ‘more inoculum does not always lead to more immune protection’. The modeling and analysis framework presented here could be applied to data from specific vaccine candidates and help to more efficiently determine the optimal dose.

## Supporting information

S1 Text Additional details and results. (DOCX)

S1 Code All R scripts and data needed to reproduce all results shown in the paper. (ZIP)

## References

[1] Ada G. Overview of vaccines and vaccination. Molecular biotechnology. 2005;29: 255–272. 10.1385/MB:29:3:255 15767703 PMC7091467

[2] Marois I, Cloutier A, Garneau É, Richter MV. Initial infectious dose dictates the innate, adaptive, and memory responses to influenza in the respiratory tract. Journal of leukocyte biology. 2012;92: 107–121. 10.1189/jlb.1011490 22504848

[3] Falsey AR. Half-dose influenza vaccine: Stretching the supply or wasting it? Archives of internal medicine. 2008;168: 2402–2403. 10.1001/archinte.168.22.2402 19064820

[4] Monath TP, Woodall JP, Gubler DJ, Yuill TM, Mackenzie JS, Martins RM, et al Yellow fever vaccine supply: A possible solution. Lancet (London, England). 2016;387: 1599–1600. 10.1016/S0140-6736(16)30195-7 27116054

[5] Crotty S, Ahmed R. Immunological memory in humans. Seminars in Immunology. 2004;16: 197–203. 10.1016/j.smim.2004.02.008 15130504

[6] Seder RA. T-cell quality in memory and protection: Implications for vaccine design. Nature Reviews Immunology. 2008;8: 486. 10.1038/nri2274 18323851

[7] R R. Bridging the knowledge gaps in vaccine design. Nature Biotechnology. 2007;25: 1361–1366. 10.1038/nbt1207-1361 18066025

[8] Pulendran B, Ahmed R. Immunological mechanisms of vaccination. Nature Immunology. 2011;12: 509–517. 10.1038/ni.2039 21739679 PMC3253344

[9] Amanna IJ, Slifka MK. Wanted, dead or alive: New viral vaccines. Antiviral Research. 2009;84: 119–130. 10.1016/j.antiviral.2009.08.008 19733596 PMC2760379

[10] Bernstein DI, Falloon J, Yi T. A randomized, double-blind, placebo-controlled, phase 1/2a study of the safety and immunogenicity of a live, attenuated human parainfluenza virus type 3 vaccine in healthy infants. Vaccine. Cincinnati Children’s Hospital Medical Center, University of Cincinnati, 3333 Burnet Avenue, Cincinnati, OH 45229, USA. david.bernstein@cchmc.org; 2011;29: 7042–7048. 10.1016/j.vaccine.2011.07.031 21782874

[11] Karron RA, Thumar B, Schappell E, Surman S, Murphy BR, Collins PL, et al Evaluation of two chimeric bovine-human parainfluenza virus type 3 vaccines in infants and young children. Vaccine. 2012;30: 3975–3981. 10.1016/j.vaccine.2011.12.022 22178099 PMC3509782

[12] Koopman G, Mooij P, Dekking L, Mortier D, Nieuwenhuis IG, van Heteren M, et al Correlation between virus replication and antibody responses in macaques following infection with pandemic influenza a virus. Journal of virology. 2015;90: 1023–1033. 10.1128/JVI.02757-15 26537681 PMC4702706

[13] Treanor J, Keitel W, Belshe R, Campbell J, Schiff G, Zangwill K, et al Evaluation of a single dose of half strength inactivated influenza vaccine in healthy adults. Vaccine. 2002;20: 1099–1105. 11803070 10.1016/s0264-410x(01)00440-6

[14] Cooper CL, Davis H, Cameron DW. Influenza vaccination with 1/10th the full dose. The New England journal of medicine. 2004;351: 2339–2340. 10.1056/NEJM200411253512219 15564552

[15] Martins RM, Maia M de LS, Farias RHG, Camacho LAB, Freire MS, Galler R, et al 17DD yellow fever vaccine: A double blind, randomized clinical trial of immunogenicity and safety on a dose-response study. Human vaccines & immunotherapeutics. 2013;9: 879–888. 10.4161/hv.22982 23364472 PMC3903908

[16] Campi-Azevedo AC, de Almeida Estevam P, Coelho-Dos-Reis JG, Peruhype-Magalhães V, Villela-Rezende G, Quaresma PF, et al Subdoses of 17DD yellow fever vaccine elicit equivalent virological/immunological kinetics timeline. BMC infectious diseases. 2014;14: 391 10.1186/1471-2334-14-391 25022840 PMC4223624

[17] Ambrose PG, Bhavnani SM, Rubino CM, Louie A, Gumbo T, Forrest A, et al Pharmacokinetics-pharmacodynamics of antimicrobial therapy: It’s not just for mice anymore. Clinical infectious diseases: an official publication of the Infectious Diseases Society of America. 2007;44: 79–86. 10.1086/510079 17143821

[18] Rhodes SJ, Zelmer A, Knight GM, Prabowo SA, Stockdale L, Evans TG, et al The tb vaccine h56+ic31 dose-response curve is peaked not saturating: Data generation for new mathematical modelling methods to inform vaccine dose decisions. Vaccine. 2016; 10.1016/j.vaccine.2016.10.060 27816373

[19] Wong S-S, Webby RJ. Traditional and new influenza vaccines. Clinical microbiology reviews. 2013;26: 476–492. 10.1128/CMR.00097-12 23824369 PMC3719499

[20] Reed G, Jewett PH, Thompson J, Tollefson S, Wright PF. Epidemiology and clinical impact of parainfluenza virus infections in otherwise healthy infants and young children <5 years old. The Journal of Infectious Diseases. 1997;175: 807–813. Available: http://www.jstor.org/stable/30129412 9086134 10.1086/513975

[21] Hall CB. Respiratory syncytial virus and parainfluenza virus. The New England Journal of Medicine. 2001;344: 1917–1928. Available: http://search.proquest.com.proxy-remote.galib.uga.edu/docview/223949760?accountid=14537 11419430 10.1056/NEJM200106213442507

[22] Lee M-S, Walker RE, Mendelman PM. Medical burden of respiratory syncytial virus and parainfluenza virus type 3 infection among US children: Implications for design of vaccine trials. Human Vaccines. 2004;1: 5–11. 10.4161/hv.1.1.1424 17038832

[23] Schmidt AC, Schaap-Nutt A, Bartlett EJ, Schomacker H, Boonyaratanakornkit J, Karron RA, et al Progress in the development of human parainfluenza virus vaccines. Expert Review of Respiratory Medicine. 2011;5: 515–526. 10.1586/ers.11.32 21859271 PMC3503243

[24] Schomacker H, Schaap-Nutt A, Collins PL, Schmidt AC. Pathogenesis of acute respiratory illness caused by human parainfluenza viruses. Current Opinion in Virology. 2012;2: 294–299. 10.1016/j.coviro.2012.02.001 22709516 PMC3514439

[25] Sato M, Wright PF. Current status of vaccines for parainfluenza virus infections. [Miscellaneous article]. Journal October 2008 2008;27 10.1097/INF.0b013e318168b76f 18820572

[26] GINSBERG HS, HORSFALL FL. Quantitative aspects of the multiplication of influenza a virus in the mouse lung; relation between the degree of viral multiplication and the extent of pneumonia. The Journal of experimental medicine. 1952;95: 135–145. 14907966 10.1084/jem.95.2.135 PMC2212055

[27] Ottolini MG, Porter DD, Hemming VG, Hensen SA, Sami IR, Prince GA. Semi-permissive replication and functional aspects of the immune response in a cotton rat model of human parainfluenza virus type 3 infection. J Gen Virol. 1996;77: 1739–1743. 10.1099/0022-1317-77-8-1739 8760420

[28] Hayden FG, Fritz R, Lobo MC, Alvord W, Strober W, Straus SE. Local and systemic cytokine responses during experimental human influenza a virus infection. Relation to symptom formation and host defense. The Journal of clinical investigation. 1998;101: 643–649. 10.1172/JCI1355 9449698 PMC508608

[29] Li Y, Handel A. Modeling inoculum dose dependent patterns of acute virus infections. Journal of theoretical biology. 2014;347: 63–73. 10.1016/j.jtbi.2014.01.008 24440713

[30] Beauchemin CAA, Handel A. A review of mathematical models of influenza a infections within a host or cell culture: Lessons learned and challenges ahead RID g-4619-2011. Bmc Public Health. 2011;11 10.1186/1471-2458-11-S1-S7 21356136 PMC3317582

[31] Smith AM, Perelson AS. Influenza a virus infection kinetics: Quantitative data and models. Wiley Interdisciplinary Reviews: Systems Biology and Medicine. 2011;3: 429–445. 10.1002/wsbm.129 21197654 PMC3256983

[32] Dobrovolny HM, Reddy MB, Kamal MA, Rayner CR, Beauchemin CAA. Assessing mathematical models of influenza infections using features of the immune response. PLoS ONE. 2013;8: e57088 10.1371/journal.pone.0057088 23468916 PMC3585335

[33] Cao P, Yan AW, Heffernan JM, Petrie S, Moss RG, Carolan LA, et al Innate immunity and the inter-exposure interval determine the dynamics of secondary influenza virus infection and explain observed viral hierarchies. PLoS computational biology. Public Library of Science; 2015;11: e1004334. 10.1371/journal.pcbi.1004334 PMC4540579 26284917

[34] Pawelek KA, Huynh GT, Quinlivan M, Cullinane A, Rong L, Perelson AS. Modeling within-host dynamics of influenza virus infection including immune responses. PLoS Computational Biology. 2012;8: 1–13. 22761567 10.1371/journal.pcbi.1002588 PMC3386161

[35] Bartlett EJ, Hennessey M, Skiadopoulos MH, Schmidt AC, Collins PL, Murphy BR, et al Role of interferon in the replication of human parainfluenza virus type 1 wild type and mutant viruses in human ciliated airway epithelium. Journal of virology. 2008;82: 8059–8070. 10.1128/JVI.02263-07 18524813 PMC2519580

[36] Potter CW, Oxford JS. Determinants of immunity to influenza infection in man. British medical bulletin. 1979;35: 69–75. 367490 10.1093/oxfordjournals.bmb.a071545

[37] Handel A, Longini IM, Antia R. Towards a quantitative understanding of the Within-Host dynamics of influenza a infections. Journal of The Royal Society Interface. 2010;7: 35–47. 10.1098/rsif.2009.0067 19474085 PMC2839376

[38] Pawelek KA, Dor D, Salmeron C, Handel A. Within-host models of high and low pathogenic influenza virus infections: The role of macrophages. PloS one. 2016;11: e0150568 10.1371/journal.pone.0150568 26918620 PMC4769220

[39] R Development Core Team. R: A language and environment for statistical computing [Internet]. Vienna, Austria: R Foundation for Statistical Computing; 2012. http://www.R-project.org/

[40] Johnson SG. The nlopt nonlinear-optimization package.

[41] Soetaert K, Petzoldt T, Setzer RW. Solving differential equations in r: Package deSolve. Journal of Statistical Software. 2010;33: 1–25. doi: 10.18637/jss.v033.i0920808728

[42] Mark Mitchell BM, al TW et. Engauge digitizer software [Internet]. http://markummitchell.github.io/engauge-digitizer/

[43] Qin L, Gilbert PB, Corey L, McElrath MJ, Self SG. A framework for assessing immunological correlates of protection in vaccine trials. J Infect Dis. Statistical Center for HIV/AIDS Research; Prevention, Fred Hutchinson Cancer Research Center, Seattle, WA, USA. lqin@scharp.org; 2007;196: 1304–1312. 10.1086/522428 17922394

[44] Plotkin SA. Vaccines: Correlates of vaccine-induced immunity. Clin Infect Dis. Sanofi Pasteur, Doylestown, Pennsylvania, USA. stanley.plotkin@sanofipasteur.com; 2008;47: 401–409. 10.1086/589862 18558875

[45] Gilbert PB, Qin L, Self SG. Evaluating a surrogate endpoint at three levels, with application to vaccine development. Stat Med. Fred Hutchinson Cancer Research Center; Department of Biostatistics, University of Washington, Seattle, WA 98109, USA. pgilbert@scharp.org; 2008;27: 4758–4778. 10.1002/sim.3122 17979212 PMC2646675

[46] Coudeville L, Bailleux F, Riche B, Megas F, Andre P, Ecochard R. Relationship between haemagglutination-inhibiting antibody titres and clinical protection against influenza: Development and application of a bayesian random-effects model. BMC Med Res Methodol. Sanofi pasteur, 2 avenue Pont Pasteur F-69367 Lyon cedex 07 France. laurent.coudeville@sanofipasteur.com; 2010;10: 18 10.1186/1471-2288-10-18 20210985 PMC2851702

[47] Feldstein LR, Matrajt L, Elizabeth Halloran M, Keitel WA, Longini IM Jr, H5N1 Vaccine Working Group. Extrapolating theoretical efficacy of inactivated influenza a/h5n1 virus vaccine from human immunogenicity studies. Vaccine. Center for Inference; Dynamics of Infectious Diseases, Fred Hutchinson Cancer Research Center, Seattle, WA, United States; Department of Biostatistics, College of Public Health; Health Professions; College of Medicine, University of Florida, Gainesville, FL, United States. Electronic address: ilongini@ufl.edu 2016;34: 3796–3802. 10.1016/j.vaccine.2016.05.067 27268778 PMC5168719

[48] Canini L, Carrat F. Population modeling of influenza A/H1N1 virus kinetics and symptom dynamics. J Virol. 2011;85: 2764–2770. 10.1128/JVI.01318-10 21191031 PMC3067928

[49] Saenz RA, Quinlivan M, Elton D, MacRae S, Blunden AS, Mumford JA, et al Dynamics of influenza virus infection and pathology. Journal of Virology. 2010;84: 3974–3983. 10.1128/JVI.02078-09 20130053 PMC2849502

[50] Ben-Shachar R, Koelle K. Minimal within-host dengue models highlight the specific roles of the immune response in primary and secondary dengue infections. Journal of the Royal Society, Interface. 2015;12 10.1098/rsif.2014.0886 25519990 PMC4305404

[51] Akondy RS, Johnson PLF, Nakaya HI, Edupuganti S, Mulligan MJ, Lawson B, et al Initial viral load determines the magnitude of the human cd8 t cell response to yellow fever vaccination. Proceedings of the National Academy of Sciences of the United States of America. 2015;112: 3050–3055. 10.1073/pnas.1500475112 25713354 PMC4364194

[52] Estcourt MJ, Létourneau S, McMichael AJ, Hanke T. Vaccine route, dose and type of delivery vector determine patterns of primary cd8+ t cell responses. European journal of immunology. 2005;35: 2532–2540. 10.1002/eji.200535184 16144036

[53] Wang Z, Kedzierski L, Nuessing S, Chua BYL, Quiñones-Parra SM, Huber VC, et al Establishment of memory cd8+ t cells with live attenuated influenza virus across different vaccination doses. The Journal of general virology. 2016;97: 3205–3214. 10.1099/jgv.0.000651 27902386

