# Supplementary material for: Exploring the impact of inoculum dose on host immunity and morbidity to inform model-based vaccine design
Source: PLoS Comput Biol. 2018 Oct 1;14(10):e1006505. doi: 10.1371/journal.pcbi.1006505 (PMC6181424; doi:10.1371/journal.pcbi.1006505)
Supplement: S1 Text — (DOCX) [file pcbi.1006505.s001.docx]

Exploring the impact of inoculum dose on host immunity and morbidity to inform model-based vaccine design

2018-08-06

# Authors

- Andreas Handel^1^, Department of Epidemiology and Biostatistics, The University of Georgia, Athens, GA, USA
- Yan Li, Institute of Bioinformatics, The University of Georgia, Athens, GA, USA. Current address: Center for Research Informatics, University of Chicago, Chicago, IL, USA
- Brian McKay, Department of Epidemiology and Biostatistics, The University of Georgia, Athens, GA, USA
- Kasia A. Pawelek, Department of Mathematics and Computational Science, University of South Carolina Beaufort, Bluffton, SC, USA
- Veronika Zarnitsyna, Department of Microbiology and Immunology, Emory University School of Medicine, Atlanta, GA, USA
- Rustom Antia, Department of Biology, Emory University, Atlanta, GA, USA

^1^ corresponding author: [ahandel@uga.edu](mailto:ahandel@uga.edu)

# Overview of SM materials

This document briefly describes all supplied files and contains some additional results.

# Files provided as SM materials

Files are provided to reproduce all results. The files are in different folders inside a zipped file. The contents of the files are as follows:

The ‘IAV data’ folder contains all files to produce the results for the IAV data.

- The ‘fit-iav-model.R’ file fits the model to the IAV data stored in ‘ginsberg52.csv’.
- The ‘simulate-iav-model.R’ file uses the best fit parameter estimates and runs simulations of the infection for a range of inoculum doses.
- The ‘plot-iav-results.R’ file loads results produced by the other 2 scripts and creates figures.
- The ‘iavequations.R’ file contains the set of differential equations for the model and is called by the other scripts.
- All scripts load from and save results to the ‘resultfiles’ folder.

The folder ‘HPIV data’ has the exact same types of files as the ‘IAV data’ folder, with ‘iav’ replaced by ‘hpiv’. These scripts fit the HPIV data, run simulations and produce figures, in complete analogy to the IAV scripts. The IAV files are somewhat better commented.

The folder ‘IAV vaccine’ has a subset of the files of the other folders. They contain the scripts that run the model and produce figures. There is no data and no fitting.

The folder ‘other scripts’ contains scripts and data for the remaining figures of the paper. Specifically:

- The file ‘morbidity-fit.R’ loads the data from Hayden et al. 1998, processes it and fits a sigmoid function to scaled innate response and symptom score.
- The file ‘plot-protect-morbidity-ratio.R’ produced the protection/morbidity ratio plots shown in the paper.

The folder ‘resultfiles’ will contain all the results (data and figures) produced by the various scripts. Note that some of these files are also loaded and further processed by several of the R scripts. To run everything, execute the ‘other scripts’ first, after that you can execute IAV, HPIV and vaccine in any order. For IAV and HPIV, the best-fit is already saved in the ‘resultfiles’ folder. You can still run the fitting script, which loads the previous best fit file and uses it as a starting point. All other result files are produced by running the scripts described above.

## Best fit parameter values

Table 1 shows the parameter estimates for the best model fits. Note that the model is over-determined, and as such the best fit estimates for the parameter values are highy uncertain (sloppy). For our situation, where the focus is on a model that describes the data well, and not on estimating parameters, we deem this possible over-fitting acceptable [1,2], but provide the caveat that the best fit parameter values provided in the table should not be considered robust estimates of biologically meanigful quantities.

Table 1 Best fit parameter values. All rates are in units of inverse hours. The virus initial conditions have units matching those of the data. Parameters *b*, *p*, *k_A_*, *h_V_* and *h_F_* have, in addition to time units, units of (inverse) virus to ensure all model terms have the correct units.

| Virus | IAV | HPIV |
| --- | --- | --- |
| V1 | 3.03e+03 | 1.06e+03 |
| V2 | 6.07e+02 | 1.30e+02 |
| V3 | 2.20e+01 | 1.10e+01 |
| V4 | 9.18e-02 | 7.84e+00 |
| V5 | 3.92e-03 | 9.00e-05 |
| b | 6.31e-05 | 8.61e-07 |
| bp | 6.03e-13 | 7.73e-13 |
| kA | 9.91e-05 | 1.90e-05 |
| kAp | 5.82e+00 | 2.99e-07 |
| dI | 1.00e-03 | 2.17e-02 |
| dV | 1.21e+02 | 4.06e-02 |
| dA | 4.00e-03 | 2.94e-07 |
| p | 5.08e-02 | 2.62e+03 |
| gF | 4.45e-01 | 7.24e-02 |
| sF | 4.78e-10 | 3.24e+05 |
| hV | 1.79e+01 | 1.80e+01 |
| hF | 1.54e-02 | 2.96e+00 |
| Fmax | 1.00e+10 | 1.09e+03 |
| rA | 1.89e-02 | 1.00e+03 |
| gB | 6.32e-02 | 4.46e-02 |

## Optimal Inoculum dose illustration

Once immune protection and morbidity as a function of inoculum dose are predicted by the model, one can potentially determine optimal inoculum dose choices. As discussed in the main text, the optimal amount depends on the main goals of the vaccine formulation. One could, for instance, choose a minimum acceptable level of immune protection or maximum acceptable level of morbidity, and determine the inoculum dose for those criteria. Another possibility is to compute and maximize a quantity that is a compound of immune protection and morbidity, with specific weights assigned to protection and morbidity. We illustrate this idea conceptually by looking at a very simple quantity, namely the ratio of immune protection to morbidity (as defined by the area under the curve), P/M. Figure 1 illustrates this quantity for the IAV and HPIV infections as well as for the inactivated vaccine.


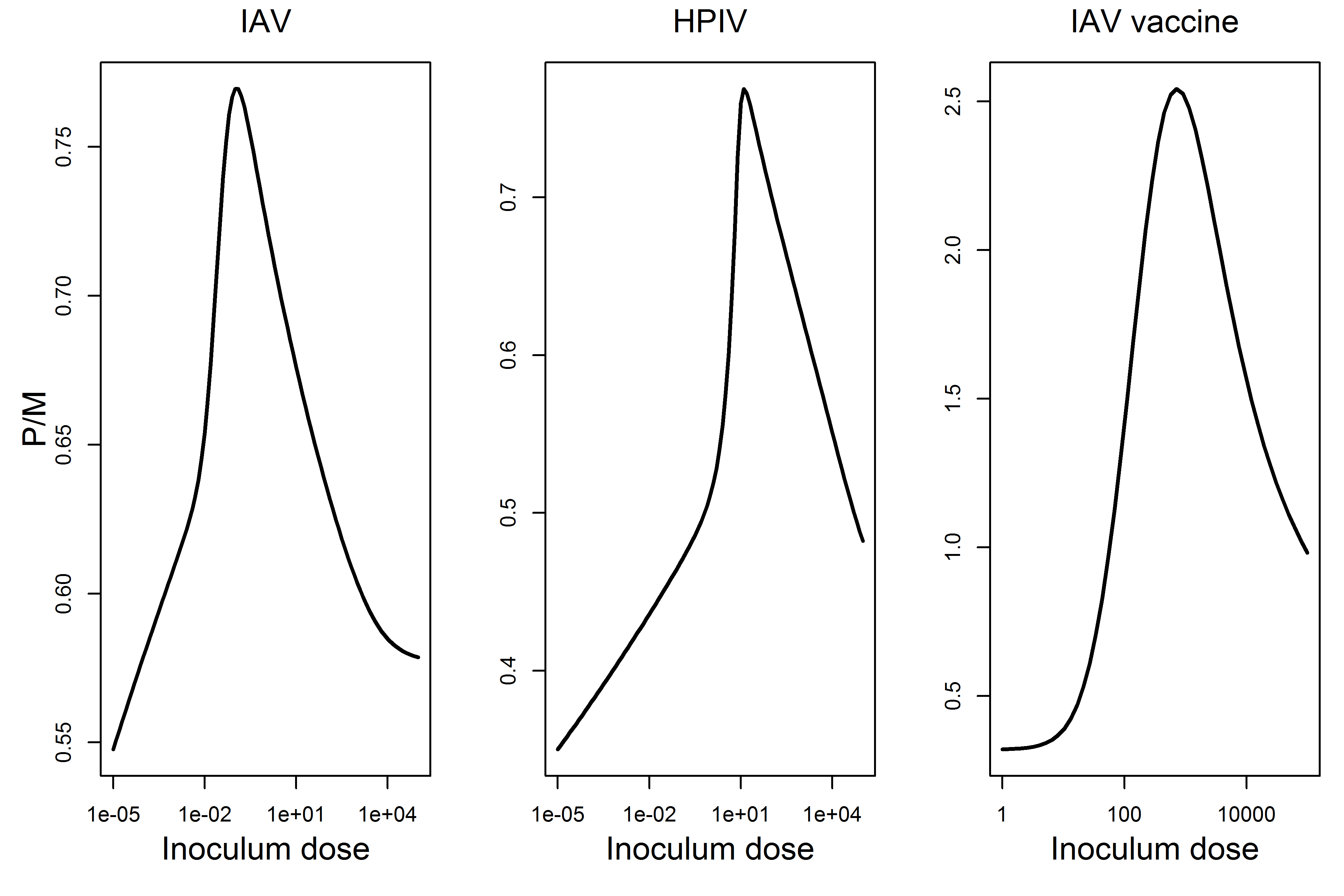


Figure 1 Ratio of protection, P, over morbidity, M, for different inoculum doses.

In each case, the amount of inoculum that leads to the highest ratio of P/MAUC occurs at an intermediate dose.

# References

1. Gutenkunst RN, Waterfall JJ, Casey FP, Brown KS, Myers CR, Sethna JP. Universally sloppy parameter sensitivities in systems biology models. PLoS Comput Biol. Laboratory of Atomic; Solid State Physics, Cornell University, Ithaca, New York, USA. rng7@cornell.edu; 2007;3: 1871–1878. doi:[10.1371/journal.pcbi.0030189](https://doi.org/10.1371/journal.pcbi.0030189)

2. White A, Tolman M, Thames HD, Withers HR, Mason KA, Transtrum MK. The limitations of model-based experimental design and parameter estimation in sloppy systems. PLoS computational biology. 2016;12: e1005227. doi:[10.1371/journal.pcbi.1005227](https://doi.org/10.1371/journal.pcbi.1005227)
